# Ultrasound‐guided motor unit scanning electromyography

**DOI:** 10.1002/mus.27720

**Published:** 2022-10-10

**Authors:** Stuart Maitland, Julie Hall, Andrew McNeill, Ben Stenberg, Ian Schofield, Roger Whittaker

**Affiliations:** ^1^ Translational and Clinical Research Institute, Henry Wellcome Building for Neuroecology Newcastle University Newcastle upon Tyne UK; ^2^ Newcastle Upon Tyne Hospitals NHS Trust Newcastle upon Tyne UK

**Keywords:** biomarker, motor unit territory, scanning EMG, ultrasound

## Abstract

**Introduction/Aims:**

Measuring the spatial dimensions of a single motor unit remains a challenging problem, and current techniques, such as scanning electromyography (EMG), tend to underestimate the true dimensions. In this study we aimed to estimate more accurately the dimensions of a single motor unit by developing a clinically applicable scanning EMG protocol that utilizes ultrasound imaging to visualize and target a transect through the center of a single motor unit.

**Methods:**

Single motor unit twitches in the tibialis anterior muscles of healthy volunteers were elicited via stimulation of the fibular nerve, visualized with ultrasound, and targeted with an intramuscular EMG electrode. The electrode was moved by hand in small steps through the motor unit territory. Ultrasound video output was synchronized to EMG capture, and the needle position was tracked at each step.

**Results:**

Eight recordings from six participants were collected. The technique was quick and easy to perform (mean time, 6.1 minutes) with reasonable spatial resolution (mean step size, 1.85 mm), yielding motor unit territory sizes between 1.53 and 14.65 mm (mean, 7.15 mm).

**Discussion:**

Ultrasound‐guided motor unit scanning EMG is a quick and accurate method for obtaining a targeted motor unit transect. This combination of two readily available clinical tools provides insights into the dimensions and internal structure of the motor unit as a marker for neuromuscular conditions.

**Abbreviations**
EMG: electromyography
MRI: magnetic resonance imaging
MU: motor unit
MUP: motor unit potential
TA: tibialis anterior

## 1 INTRODUCTION

Conventional needle electromyography (EMG) records the motor unit potential (MUP), which is the spatially summated electrical activity from muscle fibers within the vicinity of the needle tip. With appropriate filter settings, information on local fiber density can be obtained, but the dimensions of the motor unit (MU) and its internal structure cannot be determined. Several techniques have been developed that are aimed to address this lack of spatial information, including multielectrodes,^1^, ^2^, ^3^ paired needle insertions,^4^ and, more recently, MU magnetic resonance imaging (MRI).^5^ Perhaps the best known of these is scanning EMG,^6^ wherein a concentric needle electrode is withdrawn back through an MU in fixed increments to build a continuous representation of electrical activity within a corridor of the MU.

Scanning EMG has been used to measure MU territory in muscular dystrophy^7^ as well as MU territory and temporal dispersion in neurogenic and myogenic disease.^8^ Most studies using scanning EMG demonstrated no changes in MU territory size in either myogenic^7^, ^9^ or neurogenic disease, with one exception that showed a significant increase in the tibialis anterior (TA) muscle in patients with neurogenic disease.^8^ In contrast, notable internal structural changes within the MU have been shown in both disease groups, including fragmentation and increased polyphasic segments.^10^

Scanning EMG has some limitations. First, it requires the insertion of two needles into the muscle and specialized equipment (computer‐controlled stepper motor), increasing the time taken yet limiting its availability. Second, it does not consider skin elasticity or the stiction of the muscle surrounding the needle. This means that, although the stepper motor may produce a defined and highly accurate step size in relation to the skin surface, there is no guarantee that the needle tip will have moved by the equivalent amount in relation to the MU. Tissue stiction may be altered in neuromuscular diseases due to intramuscular fat infiltration, fibrosis, or increased fiber density, potentially increasing this error.

The most fundamental limitation of both scanning EMG and multielectrode techniques is that the needle is inserted “blind,” and the trajectory of the needle through the MU remains unknown. Assuming a circular MU cross‐section, the path of the needle is a chord to that circle. If performed blindly, a linear path of a needle through the unit may be anywhere between two tangents to the circle (Figure S1). Therefore, the median random EMG scan through a circular MU will underestimate the maximal diameter by a factor of ~0.87.

This is further complicated by the shape of the MU territory, which, based on histological data in animals, may be oval‐^11^ or crescent‐shaped.^12^ More recent data using MU MRI in humans revealed even more complex MU outlines, including spider‐shaped and split.^5^ These more complex MU outlines are likely to be underestimated to an even greater degree than the idealized circular outline.

In this study we sought to overcome these problems by using concurrent ultrasound imaging to target a single concentric EMG needle through the center of an MU, and to directly measure the step sizes as the needle was withdrawn, as this modality has the spatial and temporal resolution to resolve single MU activity.^13^, ^14^

## 2 METHODS

Ethics approval for this investigation was obtained from the research ethics committee of the Newcastle University Faculty of Medical Sciences (reference no. 2130/12840). Participants were recruited from a local volunteer pool and faculty staff members. Informed written consent was obtained from all participants before the study began. Exclusion criteria were history of a bleeding disorder, use of anticoagulants, implanted pacemaker or cardiac defibrillator, and neurological or neuromuscular conditions.

### 2.1 Experimental setup

Peripheral nerve stimulation was via a constant current bipolar stimulator (Model DS5; Digitimer, Welwyn Garden City, UK). Stimulation was a square pulse of width 0.3 to 3 milliseconds with a current range of 0 to 10 mA, delivered at 2 Hz. Pulse width and current were adjusted to selectively activate a single MU twitch. EMG activity was sampled at 10 kHz, and amplified (gain, 500 or 1000) using an EMG amplifier (Model D440; Digitimer) with a high‐pass filter at 3 Hz and low pass at 10 kHz, with an inline mains noise eliminator (HumBug; Quest Scientific, Canada), and sampled using a data acquisition unit (Micro1401; Cambridge Electronic Design, UK), which also triggered the bipolar stimulator (Figure S2).

Ultrasound guidance was achieved using an ultrasound machine with an eL18‐4 linear transducer (EPiQ; Philips Europe, Best, The Netherlands), with a frequency of 2 to 20 MHz. The video signal from the ultrasound machine was captured using a video‐capture device sampling at 60 Hz (Elgato HD60; Corsair Gaming, Fremont, California), with the clock signal from the data acquisition unit connected to the auxiliary sound input of the video‐capture device to synchronize ultrasound images with EMG and nerve stimulation.

### 2.2 Procedure

Subjects lay reclined and fully relaxed on an examination couch. The skin surface was cleaned using alcohol wipes, and skin preparation was used to improve conductivity at stimulating electrode sites (NuPrep; Weaver and Company, Denver, Colorado). Surface electrodes (50‐mm Covidien H34SG; Medtronic, Dublin, Ireland) were placed at the fibular head to stimulate the fibular nerve.^15^ The ultrasound probe was placed on the anterolateral surface of the lower leg, directly above the tibialis anterior muscle, approximately one third the distance from the tibial plateau, with the probe oriented perpendicular to the muscle. Electrical stimulation was gradually increased to elicit a visible single MU twitch. Our criteria for single MU activity on ultrasound were based on those used to determine single MU electrical activity, including:

1. Motion must be synchronous to electrical stimulation.
2. Motion must occur within a spatially discrete and consistent region of the image, without overlapping into other regions of activity.
3. Motion must appear and disappear in an all‐or‐nothing manner depending on stimulation intensity and show alternation when close to threshold.

For criterion 1, it was occasionally necessary during targeting to adjust the stimulation frequency to distinguish vascular pulsation from MU twitches. The stimulation frequency was then returned to 2 Hz for the duration of the experiment.

**FIGURE 1:**
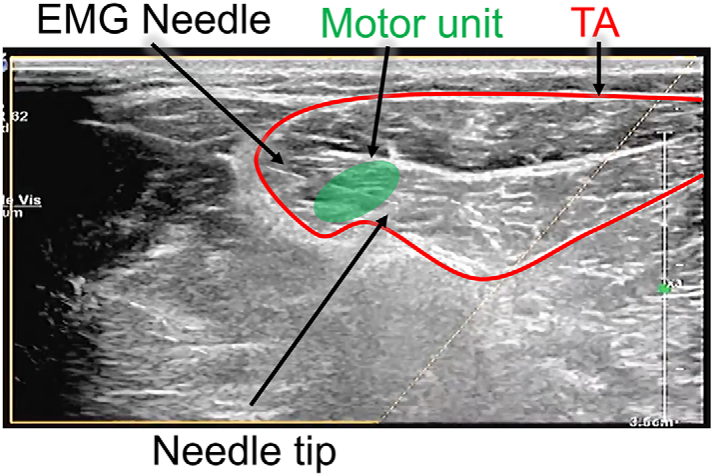
Needle in situ (white linear structure from top left to labeled needle tip) in a motor unit within tibialis anterior (TA). The needle has been targeted through the center of the motor unit and advanced through it until the motor unit potential is no longer detectable, before being withdrawn along the same trajectory by hand. For a video demonstrating the movement of the needle through the muscle, see Data S1.

The single MU twitch was targeted using a 26‐gauge concentric EMG needle (Teca Elite; Natus, Middleton, Wisconsin) under ultrasound guidance, with the needle's placement within the TA confirmed directly by ultrasound. For the muscle and needle to be visualized simultaneously, the needle was inserted at an angle of between 30° and 45°, in‐plane with the probe. The needle was deliberately targeted toward the center of the region of movement (Figure 1; Data S1 for video of needle targeting and withdrawal).

The needle was advanced through the center of the MU and out its other side, beyond the region of visible twitch. The needle was then withdrawn in increments as small as the operator could make and held in each position for 20 stimuli. Before each needle movement, the time of movement was marked on the EMG recording using synchronous keyboard input for later determination of position.

Post hoc, all data were analyzed using MATLAB 2019a (The Mathworks, Natick, Massachusetts). To correct for movement of the ultrasound probe or distortion of the muscle tissue, the needle tip coordinates (*x, y*) in each step in needle position were labeled on‐screen by a human operator (S.M.), as were the *x, y* coordinates of the edges of three fascial planes, which acted as landmarks. Between steps, the *x, y* change of each landmark position was measured, and the mean change was used to compensate the needle tip coordinates for this change. In this way, the needle position would be corrected for probe drift or tissue distortion according to:

$$∆{\text{Landmark}}_{i}=\frac{\sum {\text{Landmark}}_{i}-{\text{Landmark}}_{i+1}}{3}$$

$$\text{Corrected}\;{\text{needle position}}_{i}={\text{needle position}}_{i}-∆{\text{Landmark}}_{i}$$

The EMG traces were analyzed offline to measure the spatial extent of each MU. This was defined as the positions of the outermost traces that demonstrated synchronous MUP activity greater than 50 μV, as done in scanning EMG.^16^ MU parameters, including amplitude and duration, were marked manually on unrectified, unaveraged traces in each position, with the amplitude defined as the maximum peak‐to‐peak amplitude at any position within the MU, and the duration defined as the longest duration of any position within the MU.^7^

### 2.3 Modeling MU dimensions

We built simulations to exhaustively estimate the chord length of different two‐dimensional MU shapes, as found in imaging of human MUs.^5^ We drew approximately 500 × 500 pixel shapes (circle, wide ellipse, narrow ellipse, pennate) and measured their chord lengths through every possible chord in 1‐pixel increments, in every orientation, rotating the shape about its origin in 1‐degree steps. Where the chord passed through two borders (eg, the limbs of the pennate shape), the gap between was discounted; the length was considered the sum of the chord lengths of limb 1 + limb 2. We compared the median lengths of those chords that traversed the center of the shape (simulating needle targeting) vs those that did not (simulating “blind” needle insertion).

## 3 RESULTS

### 3.1 Modeling

Our modeling confirmed the theoretical underestimate of 86% median diameter for a circular MU territory (Table 1). We also confirmed that any deviation from a circular outline increased this error, with the median diameter of “narrower” shapes underestimating the true maximal diameter much more than “wider” shapes. Modeling targeting the needle through the center of the MU reduced this error for every shape tested; for instance, 74% for a wide ellipse targeted vs 60% blind.

**TABLE 1:** Simulated motor unit diameters measured exhaustively in every orientation

Shape	Example	Untargeted^a^	Targeted^b^
		median underestimate	median underestimate
Circle	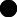	86%	100%
Wide ellipse	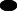	60%	74%
Narrow ellipse	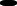	28%	33%
Pennate	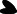	56%	62%

*Note*: Results are standardized measures to the maximal diameter (ie, 100%). ^a^Refers to transects through every position in the motor unit. ^b^Refers only to transects that traverse the center point of the unit.

### 3.2 Practical aspects

Eight recordings were collected from six individuals 29 to 49 years of age (Table 2). The mean time from needle placement to withdrawal was 6.1 minutes. There was a clear learning effect, with the first recording taking 12 minutes and the last only 3 minutes. Only minor discomfort was described by participants, mostly during initial needle insertion. Only minor, self‐resolving bleeding was experienced, consistent with a routine clinical EMG recording. We discovered during preliminary experiments that conduction between the ultrasound gel and the needle shaft could occur, resulting in a direct current offset that saturated the amplifier. This was resolved by covering the ultrasound probe and gel with a surgical probe cover.

**TABLE 2:** Measured parameters of single motor unit scans

ID	Stimulation current (mA)	Experiment duration (min)	Transect length (mm)	Transect length (steps)	Onset latency (ms)^a^	MUP duration (ms)^b^	Max MUP amplitude (μV)
1	3.64	12:07	7.82	5	37	5.60	134
2	3.87	07:54	11.29	8	37	5.80	200
3	7.95	06:38	1.53	4	6.8	5.27	190
4	6.58	05:04	8.80	6	9.6	10.20	250
5	6.99	04:05	3.27	3	8.9	9.40	520
6	3.40	06:34	8	3	4.3	7.90	237
7	5.50	02:59	7	5	5.3	7.90	101
8	2.60	03:33	9.50	4	4.8	2.80	119
Mean	5.07	06:06	7.15	4.75	14.2	6.86	219

Abbreviations: ID, identification number; Max, maximum; MUP, motor unit potential.

^a^ Earliest onset of any position with valid MUP (amplitude >50 μV).

^b^ Longest duration of any valid MUP.

Stimulation currents to achieve single MU activation ranged from 2.6 to 7.9 mA. The mean step size of the needle between positions across all recordings was 1.85 (standard deviation [SD], 1.07) mm. For individual recordings, the mean step size ranged from 1.03 to 2.91 mm. Between three and eight positions (median, 4.5) were recorded from within each MU.

### 3.3 MU parameters

**FIGURE 2:**
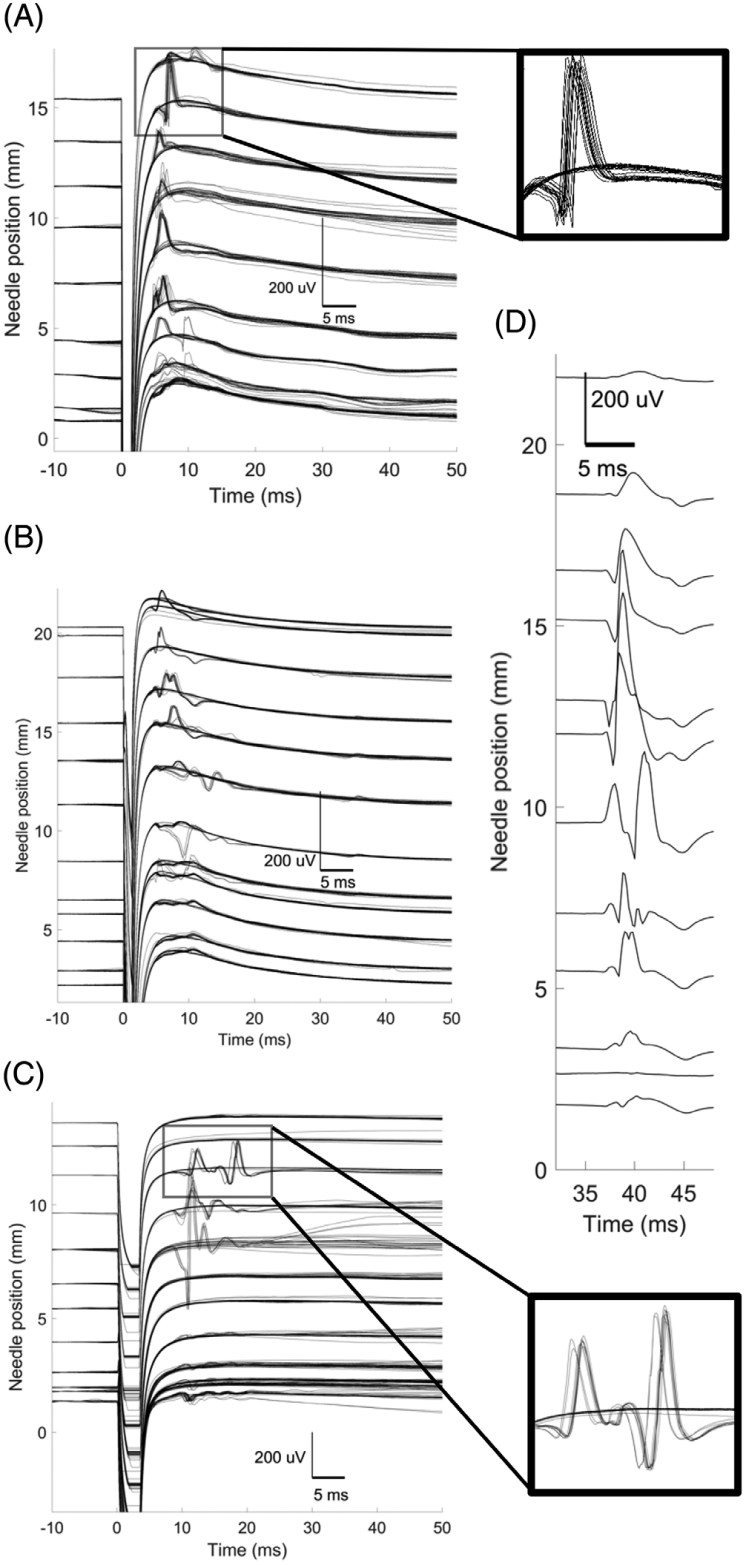
Example electromyographic scans from healthy human tibialis anterior muscle. For each scan, the Y origin indicates the position of the needle from the deepest starting position; a higher Y value indicates a shallower position. Stimulus artifact can be seen at time = 0. Each trace (n = 20) is shown at each position. A, Diffuse cannula potential at deeper positions giving way to triphasic motor unit potential (MUP), inset to highlight alternation and MUP shape (recording ID = 8). B, Evolving activity within the motor unit (recording ID = 7). Both the deep and superficial boundaries are included, permitting the motor unit territory to be estimated. C, Example of both alternation and MUP latency variability (recording ID = 4). D, Enhanced view of a single motor unit transect, with traces at each position averaged (recording ID = 2).

Quantitative analysis data of MUPs are presented in Table 2. The appearance of the MUP changed as the needle traversed the MU, with a diffuse inverted “cannula potential” at deeper positions giving way to a classic triphasic MUP at more superficial positions (Figure 2). Alternation was demonstrated, with either a consistent MUP outline or a flat trace seen at each needle position (Figure 2A). The MU territory sizes varied widely, ranging from 1.53 to 14.65 (mean, 7.15) mm. MUP durations varied from 2.8 to 10.2 milliseconds. The latency measurement was consistent within a given transect, but varied widely between recordings from 4.3 to 37 milliseconds. The maximal MU amplitudes of each transect ranged from 101 to 520 μV (mean, 219 μV) and were always located toward the center of the MU, that is, never at the furthest extent.

## 4 DISCUSSION

In this pilot study we used ultrasound to guide a needle EMG transect of single MUs in a small sample of healthy adults. Previous studies have reported a wide range of normal human MU dimensions, with mean territories ranging from 3.7 to 11.3 mm (Figure S3). Comparing our results with data obtained using scanning EMG from tibialis anterior, we found that our mean territory of 7.15 mm is well within the range obtained using this method. This is despite our use of electrical stimulation to activate MUs in contrast to voluntary activation (which activates smaller MUs first) in scanning EMG.

By directly visualizing the MU, we could ensure that the needle included the entire MU transect, compared with some published images of MU transects showing MUP activity extending to the edge of the scan.^17^

Direct visualization also allows the center of the MU to be targeted, reducing the variability in measured motor unit dimensions, as demonstrated in our modeling. In practice, we found that the positioning of the ultrasound probe and the need to achieve good needle visualization necessitated a relatively fixed trajectory of 30° to 45° to the skin surface, which meant that we could not always traverse the MU along its longest axis. Alternative needles used for EMG‐guided muscle injection may be suitable to counter this in the future, as they are designed with indentations, enabling visualization under ultrasound at more oblique angles.

One clear limitation of our technique is the relatively large step size (1.85 mm) compared with scanning EMG (50 μm), and the proportionately fewer (3 to 8) traces within the MU.

Our experience during the experiment was that there was clear stiction of the needle to the surrounding muscle tissue. This resulted in a nonlinear “limited elasticity” deformation of the surrounding tissue, pulling the surrounding muscle along with the needle for smaller movements, above which the muscle suddenly yielded and moved in relation to the needle. This phenomenon has been described in surgical robotic models with tissue phantoms,^18^ and may also occur during scanning EMG (Figure 2D in the study by Stålberg and Eriksson^16^). Our direct imaging of the needle tip did at least allow us to compensate for this effect; hence, although our technique permits only sparse sampling of the MU transect, the precise location of those samples is known.

Our aim was to develop a quick and accurate means of estimating MU dimensions and structure using readily available clinical equipment. We achieved this in some respects, in that with minimal training we were able to obtain single MU transects in a few minutes. Our technique has the advantage over scanning EMG of targeting the center of the MU, thereby producing a more accurate estimate of the MU dimensions, albeit at the expense of significantly reduced information as to its internal structure. In its current iteration, our experimental setup remains rather complex and cumbersome, particularly the external synchronization of the EMG recordings with the ultrasound video output. However, with the increased availability of EMG machines with integrated ultrasound capability, there is no fundamental reason why a single system incorporating all these features could not be developed.

## FUNDING INFORMATION

NIHR Newcastle Biomedical Research Centre, Grant Number: BH180986; Medical Research Council CARP fellowship, grant number: MR/T005483/1 (to J.H.); Medical Research Council, Confidence in Concept Newcastle Award (to R.G.W.)

## CONFLICT OF INTEREST

None of the authors have any potential conflicts of interest to disclose.

## ETHICAL PUBLICATION STATEMENT

We confirm that we have read the Journal's position on issues involved in ethical publication and affirm that this report is consistent with those guidelines.

## Supporting information


**FIGURE S1** Effect of needle path on estimated motor unit dimensions. The true maximal motor unit dimension (diameter, ⌀), as measured by a perfect bisect of the motor unit with the electromyography needle, is underestimated by every other chord through the motor unit (crd) for which *d* > 0.

**FIGURE S2** Experimental setup diagram. CED 1401 sequencer‐controlled stimulation and sampled electromyography (EMG). DS5 stimulator provided peripheral nerve stimulation of fibular nerve. EMG was sampled by D440 amplifier, filtered, and mains noise removed with HumBug. Video output from ultrasound scanner was synchronized with EMG sampling via the 1401 clock signal, input as audio‐ to the video‐capture device. Inset shows stimulating electrodes over the fibular nerve at the fibular head and ultrasound probe placed over the tibialis anterior muscle. Needle was inserted at the indicated red cross, in‐plane with the ultrasound probe.

**FIGURE S3** Motor unit territory dimensions collected from the literature. Diamonds indicate mean, and left or right arrows indicate when a mean range was specified. Lines demonstrate standard deviation, and whiskers indicate range. Abbreviations: BB, biceps brachii; EDC, extensor digitorum communis; TA, tibialis anterior. References legend: *Maximum Feret diameter; ^❖^Personal communication in addition to published data.

**DATA S1** Needle insertion and withdrawal.mp4. Video shows ultrasound‐guided needle targeting, demonstrating the single motor unit twitch (center of screen), insertion of needle through center of motor unit, and stepwise withdrawal. File available at: https://github.com/stuartbman/ultramusevideos.

**APPENDIX S1** Supporting Information.

## Data Availability

The data that support the findings of this study are available from the corresponding author upon reasonable request.
