# Supplementary figures and images for: Ultrasound‐guided motor unit scanning electromyography
Source: Muscle Nerve. 2022 Oct 10;66(6):730–5. doi: 10.1002/mus.27720 (PMC9828660; doi:10.1002/mus.27720)

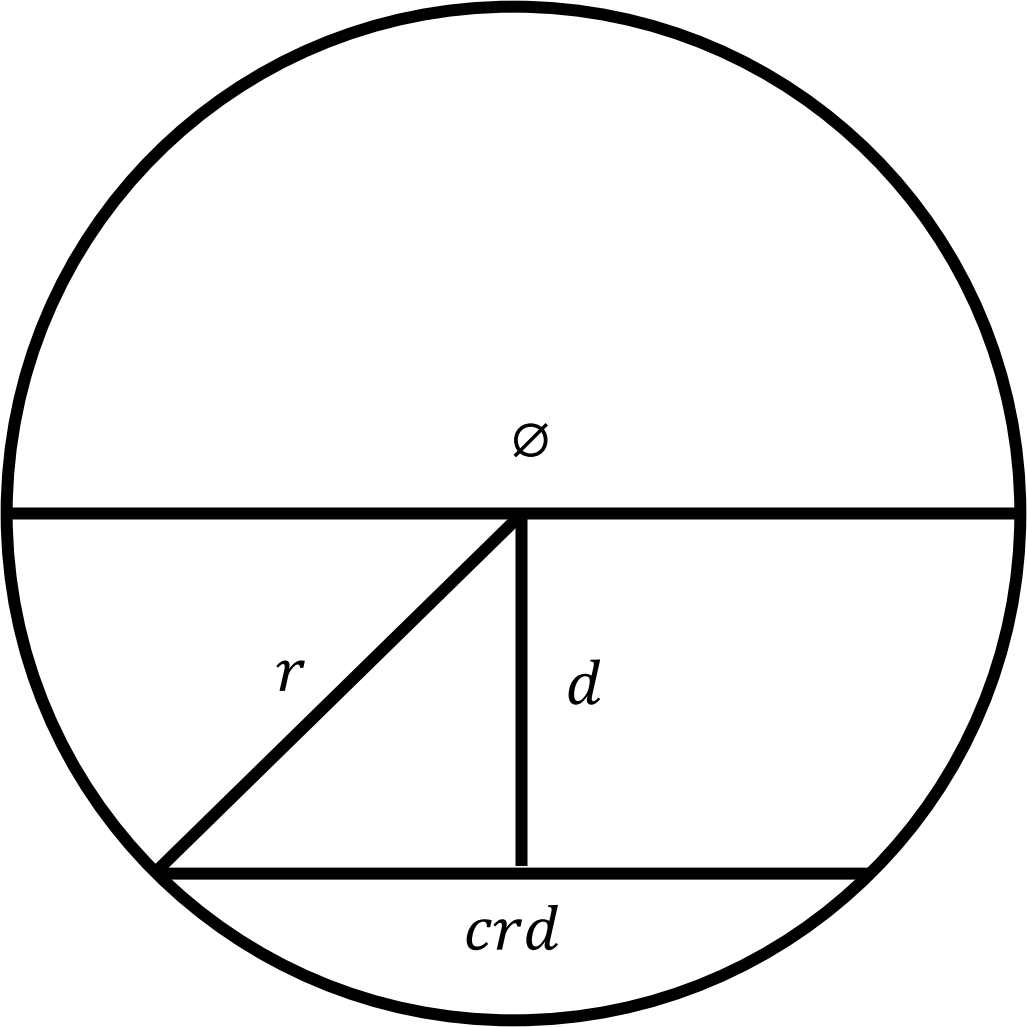

Supplement: Supplementary file 1 — FIGURE S1 Effect of needle path on estimated motor unit dimensions. The true maximal motor unit dimension (diameter, ⌀), as measured by a perfect bisect of the motor unit with the electromyography needle, is underestimated by every other chord through the motor unit (crd) for which d > 0. [file MUS-66-730-s004.pdf]

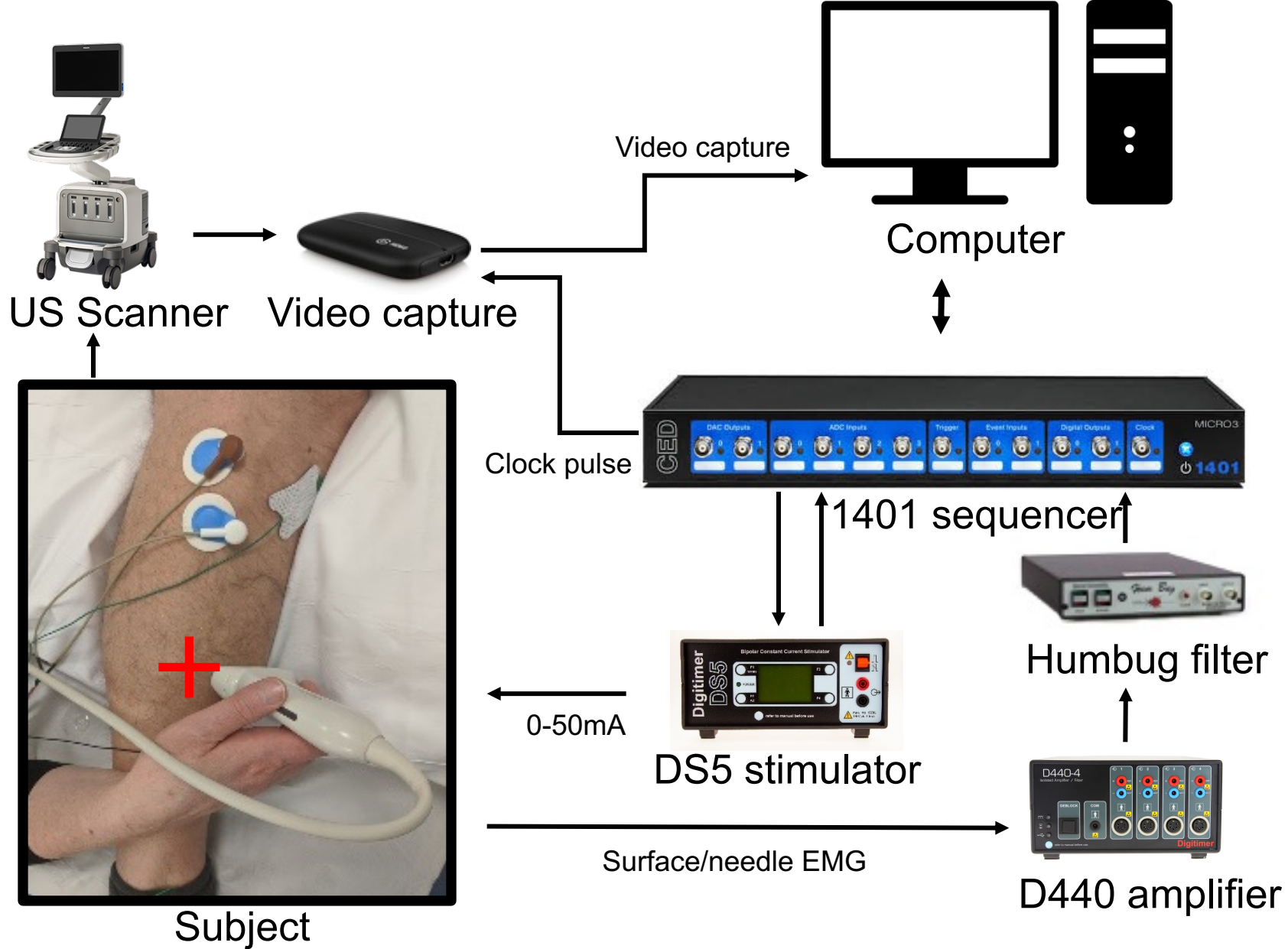

Supplement: Supplementary file 2 — FIGURE S2 Experimental setup diagram. CED 1401 sequencer‐controlled stimulation and sampled electromyography (EMG). DS5 stimulator provided peripheral nerve stimulation of fibular nerve. EMG was sampled by D440 amplifier, filtered, and mains noise removed with HumBug. Video output from ultrasound scanner was synchronized with EMG sampling via the 1401 clock signal, input as audio‐ to the video‐capture device. Inset shows stimulating electrodes over the fibular nerve at the fibular head and ultrasound probe placed over the tibialis anterior muscle. Needle was inserted at the indicated red cross, in‐plane with the ultrasound probe. [file MUS-66-730-s005.pdf]

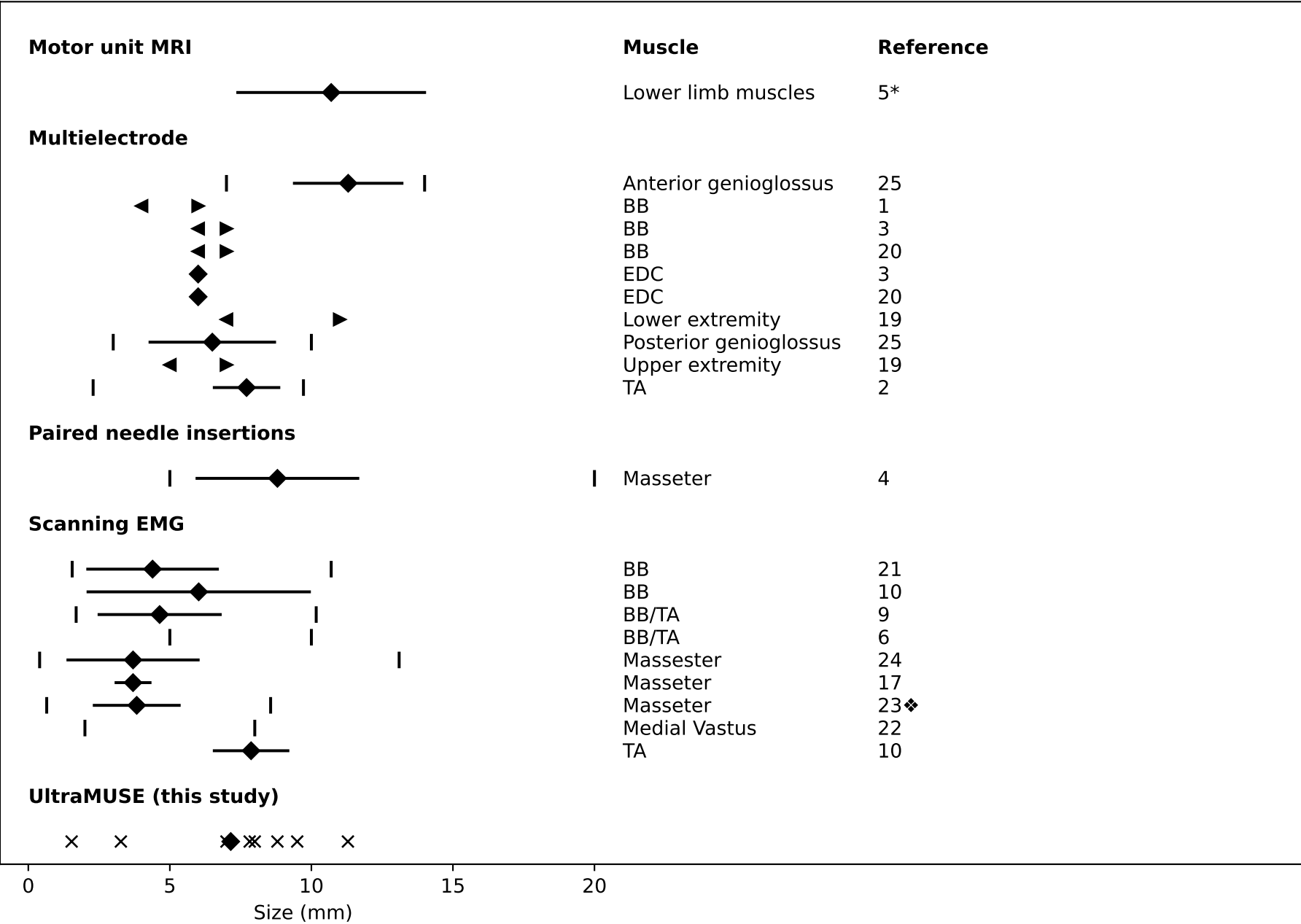

Supplement: Supplementary file 3 — FIGURE S3 Motor unit territory dimensions collected from the literature. Diamonds indicate mean, and left or right arrows indicate when a mean range was specified. Lines demonstrate standard deviation, and whiskers indicate range. Abbreviations: BB, biceps brachii; EDC, extensor digitorum communis; TA, tibialis anterior. References legend: *Maximum Feret diameter; ❖Personal communication in addition to published data. [file MUS-66-730-s002.pdf]
