# Supplementary material for: Ultrasound‐guided motor unit scanning electromyography
Source: Muscle Nerve. 2022 Oct 10;66(6):730–5. doi: 10.1002/mus.27720 (PMC9828660; doi:10.1002/mus.27720)
Supplement: Supplementary file 4 — DATA S1 Needle insertion and withdrawal.mp4. Video shows ultrasound‐guided needle targeting, demonstrating the single motor unit twitch (center of screen), insertion of needle through center of motor unit, and stepwise withdrawal. File available at: https://github.com/stuartbman/ultramusevideos. [file MUS-66-730-s001.docx]

**Supplementary File 4- needle insertion and withdrawal.mp4.** Video of ultrasound guided needle targeting, demonstrating the single motor unit twitch (centre of screen), insertion of needle through centre of motor unit, and stepwise withdrawal. File available at: https://github.com/stuartbman/UltraMUSEvideos
