# Supplementary material for: Ultrasound‐guided motor unit scanning electromyography
Source: Muscle Nerve. 2022 Oct 10;66(6):730–5. doi: 10.1002/mus.27720 (PMC9828660; doi:10.1002/mus.27720)
Supplement: Supplementary file 5 — APPENDIX S1 Supporting Information. [file MUS-66-730-s003.docx]

Supplementary Captions

**Supplementary Figure 1- Effect of needle path on estimated motor unit dimensions.** The true maximal motor unit dimension (diameter, ⌀), as measured by a perfect bisect of the motor unit with the EMG needle, is underestimated by every other chord through the motor unit ($crd$) for which d>0.

As a result, a random chord length may be anywhere between zero and the true diameter of the unit:

$2\sqrt{r^{2}-d^{2}}$ *(1)*

Where: r is the radius of the circle

d is the distance of the chord away from the centre of the circle (range $\pm r$)

$\text{⌀}$ is the diameter of the circle = 2r

Since the distribution of chord lengths is one-tailed with respect to the maximum diameter of the motor unit, the median chord length can be assumed to be at the midpoint between the diameter and a tangent at r which is at $d= \frac{r}{2}$.

$2\sqrt{r^{2}-({\frac{r}{2})}^{2}} =1.73r=0.866\text{⌀}$ *(2)*

**Supplementary Figure 2- Experimental setup diagram.** CED 1401 sequencer controlled stimulation and sampled EMG. DS5 stimulator provided peripheral nerve stimulation of fibular nerve. EMG was sampled by D440 amplifier, filtered, and mains noise removed with Humbug. Video output from ultrasound scanner was synchronised with EMG sampling via the 1401 clock signal, input as audio to the video capture device. Inset shows stimulating electrodes over the fibular nerve at the fibular head and ultrasound probe placed over the tibialis anterior muscle. Needle was inserted at indicated red cross, in plane with ultrasound probe.

**Supplementary Figure 3- Motor unit territory dimensions collected from the literature**. ◆ = mean, with ◀ ▶ where a mean range was specified. Lines demonstrate the SD, and whiskers are the range. BB=Biceps brachii, EDC = Extensor digitorum communis, TA = Tibialis anterior. References legend: *Maximum Feret diameter, ❖Personal communication in addition to published data.

**Supplementary File 4- needle insertion and withdrawal.mp4.** Video of ultrasound guided needle targeting, demonstrating the single motor unit twitch (centre of screen), insertion of needle through centre of motor unit, and stepwise withdrawal. File available at: https://github.com/stuartbman/UltraMUSEvideos
